# Reactivity Ratios of Biobased Dibutyl Itaconate with Conventional and Renewable (Meth)Acrylates: Influence of Depropagation

**DOI:** 10.1021/acs.biomac.5c01505

**Published:** 2025-10-13

**Authors:** Jyoti Gupta, Radmila Tomovska, Shaghayegh Hamzehlou, Miren Aguirre

**Affiliations:** † POLYMAT, Kimika Aplikatua Saila, Kimika Fakultatea, University of the Basque Country UPV-EHU, Joxe Mari Korta Zentroa, Tolosa Hiribidea 72, 20018 Donostia-San Sebastián, Spain; ‡ IKERBASQUE, Basque Foundation for Science, Plaza Euskadi 5, 48009 Bilbao, Spain

## Abstract

The global push for sustainability has driven interest in renewable monomers such as dibutyl itaconate (DBI). Despite its potential for biobased high-performance polymers, DBI’s copolymerization with (meth)­acrylates is challenging due to low propagation rates, depropagation at moderate temperatures, and unfavorable reactivity ratios. This study investigates the solution copolymerization of DBI with both conventional, butyl acrylate (BA) and methyl methacrylate (MMA), and biobased (2-octyl acrylate (2-OA) and isobornyl acrylate (IBOA)) monomers. Using in situ ^1^H NMR and nonlinear least-squares fitting, reactivity ratios were determined: BA/DBI (*r*_BA_ = 0.68, *r*_DBI_ = 0.68), MMA/DBI (*r*_MMA_ = 3.53, *r*_DBI_ = 0.38), 2-OA/DBI (*r*_2‑OA_ = 1.64, *r*_DB_I = 1.44), and IBOA/DBI (*r*_IBOA_ = 1.87, *r*_DBI_ = 2.0). The study also clarifies discrepancies in literature values for BA/DBI due to uncertainties in DBI’s depropagation rates. DBI copolymers with BA, 2-OA, and IBOA showed a lower composition drift than MMA/DBI, which showed a greater disparity in reactivity ratios.

## Graphical abstract

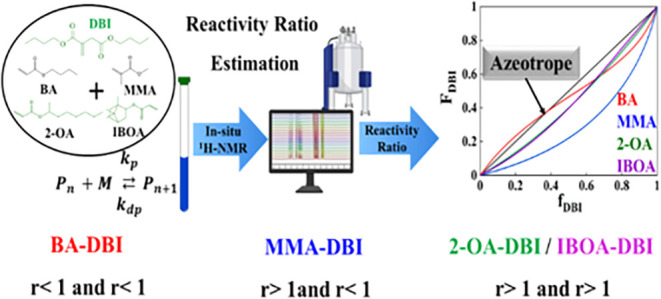

## 1 Introduction

Polymers have significantly enhanced human life; however, critical issues regarding their raw material origins and end-of-life disposal persist. Nowadays, the majority of commodity polymers are synthesized from nonrenewable petroleum feedstocks, raising sustainability challenges and having significant environmental impacts.^1–6^ Common monomers, such as methyl methacrylate (MMA) and butyl acrylate (BA) are still derived from petroleum feedstock. While biobased synthesis routes for these monomers are under active research, current methodologies are not yet economically viable for large-scale industrial applications.^7^ Therefore, one of the pathways to improve sustainability could be to replace petroleum-based monomers with biobased alternatives, which is becoming a prominent area of interest for industry and academia.^8–15^

One of the most important classes of compounds obtained from lignocellulose biomass is organic acids. Among these, itaconic acid (IA) is an important renewable feedstock obtained by the fermentation of starch originating from biomass, together with its derivatives, which are essential renewable chemicals due to their abundant availability, sustainability, and cost-effectiveness.^16–18^ From the reaction of IAs with biobased alcohols, itaconate esters with 100% biocontent can be produced.^19–24^ The global market for IA is projected to reach US$126 million by 2030, growing at a 3.9% annual growth rate.^21,25^

However, the incorporation of itaconates in radical polymerization is not straightforward. Several studies from the 1950s, conducted by researchers such as Nagai,^26^ Madruga,^27^ Fernández-García,^28^ and Ostu,^29^ made significant contributions by investigating the kinetics of itaconate esters in radical polymerization. It has been widely recognized since then that itaconate homopolymerization has limitations due to the steric effects of its bulky substituents, resulting in low monomer conversions and polymer molar masses.^27,30,31^ Consequently, the propagation rate coefficients (kp) are much lower than those for methacrylates, by more than 2 orders of magnitude.^32,33^ Even though the activation energies are similar, the reduced propagation rate is related to a reduced pre-exponential factor due to the steric hindrance.^34,35^ The slower propagation rate, therefore, increases the extent of chain transfer to solvent and monomer.^36–39^ However, these studies did not address the associated depropagation phenomena. In free-radical copolymerization, depropagation can occur at low monomer concentrations around the ceiling temperature (*T*_c_) value of one or both comonomers in the system^40,41^ (specifically in the case of sterically hindered monomers), lowering the overall polymerization rate. It can also affect the molar mass and composition of the copolymer. Szablan et al.^33^ investigated the depropagation kinetics of the itaconate ester monomers, highlighting its detrimental effect on the polymerization rate and polymer properties such as molar mass at temperatures below 100 °C. The equilibrium state related to the depropagation and propagation mechanism of itaconate ester is well explained in the literature.^42–44^ The propagation step should be expressed as an equilibrium eq 1 ^33,42,43,45^ and the effective propagation rate, denoted by *k*_p_^eff^, is given by eq 2

1 $$P_{n}+M\begin{array}{l} k_{p}\\ \Leftrightarrow \\ k_{dp} \end{array}P_{n+1}$$

2 $$k_{p}^{\mathrm{eff}}=k_{p}-\frac{k_{\mathrm{dp}}}{\left[M\right]}$$

Although homopolymerization of itaconate esters presents distinct challenges^46–50^ as mentioned above, incorporating them in combination with common monomers in radical copolymerization presents an opportunity to increase the renewable content in current formulations.^51^ Acrylates are known to polymerize more rapidly and do not undergo depropagation, unlike itaconates.^52^ In this line, Pirman et al.^53^ explored the solution copolymerization of dibutyl itaconate (DBI) with BA under semibatch, starved-feed conditions at temperatures of 50 and 80 °C. They varied initial monomer and initiator concentrations and examined how different initiators and solvents influenced polymerization rates and molar mass distributions (MWDs). Their findings indicated that incorporating up to 50 wt % DBI into the copolymer is feasible, carrying out semibatch experiments at 110 °C under industry-standard conditions. In addition, they developed a kinetic mathematical model to estimate key kinetic parameters to control copolymer composition, polymerization rate, and polymer’s microstructure. Further, Kokol et al.^54^ carried out the copolymerization of dibutyl itaconate (DBI) at a high temperature of 110 °C with BA, MMA, and styrene (St). The polymerization rate of DBI/BA copolymerization was the highest among these systems. In contrast to the polymers derived from the DBI/MMA and DBI/St systems, the resultant polymers exhibited reduced molar masses, which were attributed to higher chain transfer reactions. Additionally, the impact of incorporating DBI into an MMA/BA latex formulation synthesized via emulsion polymerization, considering the depropagation effect at different temperatures, has been studied. The study showed that, despite the challenges posed by the DBI monomer, the polymer’s biocontent was increased, achieving high conversions within short reaction times.^55^

These experimental efforts have demonstrated the potential of DBI in copolymer systems but also highlight the need for a deeper understanding and more precise control over the resulting polymer structure and properties. Therefore, it is extremely important to find proper strategies to increase the incorporation of DBI in the copolymer and control the microstructure of the polymer to reach the target final properties. To develop such control strategies for different polymer characteristics, such as copolymer composition and MMD, a predictive mathematical model of the copolymerization is needed. A knowledge of the reactivity ratios of comonomers is essential for developing the mathematical model of copolymerization.^56–60^

Only a limited number of studies have investigated the reactivity ratios of DBI copolymerized with comonomers, such as MMA and BA, and these reports demonstrate significant inconsistencies in the reported values. The MMA/DBI system was studied by Madruga et al.^27^ at 50 °C, assuming that depropagation would be insignificant at this temperature. They reported reactivity ratios of *r*_MMA_ = 1.329 ± 0.09 and *r*_DBI_ = 0.717 ± 0.11. The BA/DBI system was similarly examined by Pirman et al.^53^ using the terminal model and without taking depropagation into account at 50 °C. The results show that both BA and DBI have reactivity ratios below 1 (*r*_BA_ = 0.59 ± 0.03/*r*_DBI_ = 0.76 ± 0.08). This indicates that both monomers prefer to copolymerize rather than homopolymerize, with DBI being slightly more reactive than BA. These results have a clear contrast to the work of Drache et al.,^61^ who explicitly included depropagation effects in their determination of the reactivity ratios for the BA/DBI pair at temperatures of 60 and 80 °C. They reported significantly different reactivity ratios (*r*_BA_ = 0.5, *r*_DBI_ = 1.26) values.

In this work, the kinetics of the solution copolymerization of DBI with conventional monomers such as MMA and BA were studied using in situ ^1^H NMR at 70 °C to clarify the discrepancies in the reported reactivity ratios of DBI with BA and MMA in the literature with explicit consideration of depropagation. In addition, the impact of the uncertainty in the reported depropagation rate coefficients of DBI on the estimated reactivity ratios was investigated. On the other hand, the copolymerization of DBI with partially biobased acrylate monomers such as 2-octyl acrylate (73% biocontent, derived from castor oil) and isobornyl acrylate (77% biocontent, derived from camphene) offers a promising route to further increase the renewable content of the resulting polymers. These monomers have demonstrated potential as sustainable alternatives to conventional petroleum-based monomers such as MMA and BA.^62–64^ Therefore, they were selected for further investigation in this study. To the best of the authors’ knowledge, no literature reports are available on the reactivity ratios of dibutyl itaconate with the aforementioned biobased acrylates. For all four copolymerization systems, the data were gathered on the individual conversions as well as copolymer composition at different molar ratios of 75:25, 50:50, and 25:75. The generated kinetic data were used to estimate the reactivity ratios of these monomer systems using a nonlinear least-squares method (NLLSQ) considering the modified Mayo–Lewis equation in the presence of depropagation.

## 2 Experimental Section

### 2.1 Materials

Dibutyl itaconate (DBI, 99% purity, Sigma-Aldrich) was used as received. The monomers methyl methacrylate (MMA, purity 99.9%) and butyl acrylate (BA, purity 99%) were purchased from Quimidroga. Isobornyl acrylate (IBOA, purity 99%, TCI) and 2-octyl acrylate (2-OA, purity 98%) were synthesized following the procedure reported elsewhere.^63^ Deuterated dimethyl sulfoxide (DMSO-*d*_6_, purity 99.9%, Eurisotop) served as a solvent. The polymerizations were initiated by azobis­(isobutyronitrile) (AIBN, Sigma-Aldrich, purity 98%). Figure 1 shows the chemical structure of the biobased monomers used in this work, as well as their biocontent.

**Figure 1:**
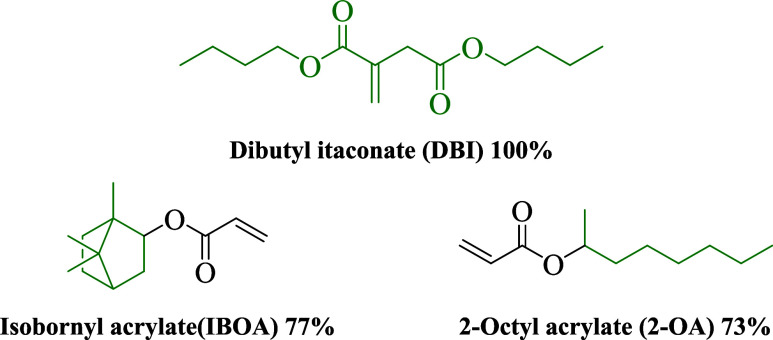
Biobased monomers, chemical structure, and their biobased content.

### 2.2 In Situ ^1^H NMR Solution Copolymerization

In situ, ^1^H NMR solution copolymerizations of BA, MMA, 2-OA, and IBOA with DBI in DMSO-*d*_6_ solvent were carried out in a Wilmad NMR tube with a length of 18 cm and a diameter of 5 mm (wall thickness of 0.43 mm) as the reaction vessel. To prevent overpressure, a hole was drilled in the lid of the NMR tube. The copolymerizations were monitored in situ. Solution polymerizations with a total monomer concentration of 30% by weight were carried out at atmospheric pressure. When the monomer solution reached 70 °C, 1 mol % (based on the monomers) of the initiator AIBN was added to the NMR tube. Three different comonomer compositions of each monomer couple were carried out at 50:50, 75:25, and 25:75 ratios.

^1^H NMR spectra were obtained by using a 500 MHz Bruker Advance NMR instrument coupled with a Z gradient broadband observe (BBO) probe. Scans for all the systems (BA/DBI, MMA/DBI, 2-OA/DBI, and IBOA/DBI) were done every 5 min for the first 30 min, every 10 min for the following 30 min, and every 30 min for the remaining 2 h 30 min. Each measurement required one scan with a relaxation delay of 10.00 s, a pulse width of 14 s, and an acquisition time of 2.23 s. Monomer conversions were calculated based on the evolution of the peaks corresponding to the vinyl protons of the monomers. For instance, BA (δ (ppm)= 5.83–5.92) and DBI (δ (ppm)= 5.74–5.78) (Figure 2). Absolute integral areas were used for the calculation of monomer conversions using eqs 3 and 4. The spectra were processed by Mestre Nova 9.0. The NMR spectra of the other three pairs (MMA/DBI, 2-OA/DBI, and IBOA/DBI) are presented in the Supporting Information (Figures S1–S3).

3 $$X_{\mathrm{Mi}}=\frac{A_{0}^{\mathrm{Mi}}-A_{t}^{\mathrm{Mi}}}{A_{0}^{\mathrm{Mi}}}$$

4 $$X_{overall}=\frac{\left(A_{0}^{M1}+A_{0}^{M2}\right)-{(A}_{t}^{M1}+A_{t}^{M2})}{A_{0}^{M1}+A_{0}^{M2}}$$

where *A*_o_^M1^ is the area of the monomer *i* at time zero, *A*_t_^M*i*^ is the area of the monomer *i* at time *t*, *X*_M*i*_ is the conversion of monomer *i*, *X*_overall_ is the total monomer conversion, *M*_1_ is monomer 1, and *M*_2_ is monomer 2.

**Figure 2:**
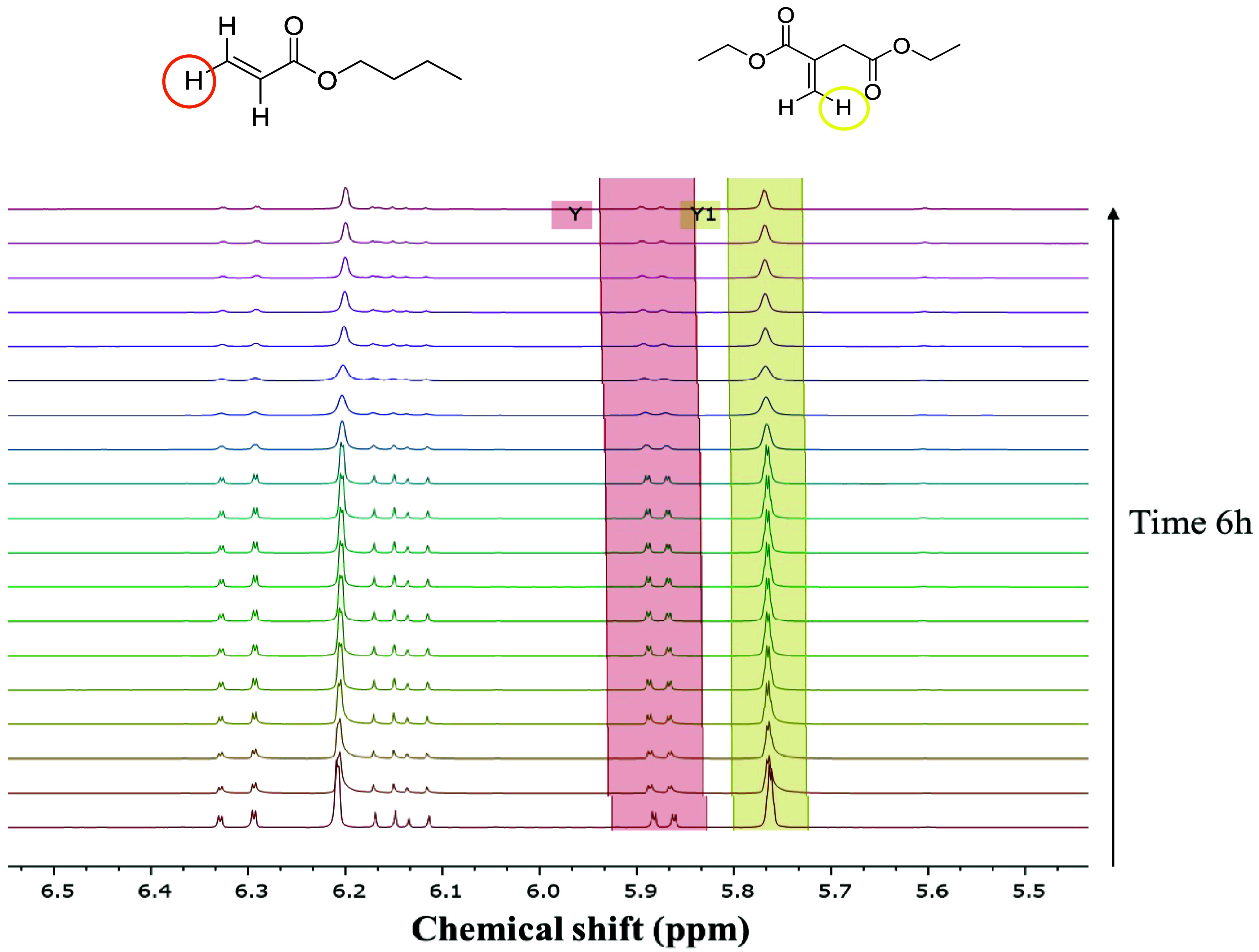
Time evolution of the proton nuclear magnetic resonance (^1^H NMR) spectra of the solution copolymerization of BA and DBI carried out at 70 °C (solids content = 30 wt % BA/DBI 50:50). The peaks that were monitored to follow the conversion of BA are marked in red (Y) and the ones for DBI are marked in yellow (Y1).

### 2.3 Estimation of Reactivity Ratios by the Nonlinear Least-Squares Method (NLLSQ)

Depropagation of one or both monomers in copolymerization will affect the monomers sequence distribution as well as copolymer composition and thus the conventional Mayo–Lewis equation^65^ will not be valid to predict the instantaneous copolymer composition of such systems.^66–68^ For a two-monomer system, Lowry^69^ developed a method to represent the impact of depropagation on the copolymer in systems where only one of the monomers has a tendency to depropagate. Here, *M*_1_ refers to the comonomers (BA, MMA, 2-OA, and IBOA) and *M*_2_ refers to DBI. Two different variations of the method were presented: Lowry Case I, where the immediate penultimate unit *M*_2_ influences depropagation, while in Case II, at least two units or a longer sequence of *M*_2_ units is required to influence depropagation. For the copolymerization of DBI with other comonomers, there are no data validating the applicability of either Lowry case I or II. However, since the Lowry case I has been used previously in the literature to model the depropagation of DBI in copolymerization,^53,61^ we have adopted Lowry case I in the present work as well. In this case, the relative rate of polymerization of each monomer is presented as follows

5 $$\frac{d\left[M_{2}\right]}{d\left[M_{1}\right]}=\frac{\left[M_{2}\right]\left(\frac{1}{1-\alpha }\right)}{r\left[M_{1}\right]+\left[M_{2}\right]}$$

where [*M*_1_] and [*M*_2_] are the concentrations of monomers *M*_1_ and *M*_2_, respectively, and α denotes the influence of depropagation in eq 6.

6 $$\alpha =\frac{\left[P_{n+1}^{2*}\right]}{\left[P_{n}^{2*}\right]}$$

A propagating radical with n units of monomer 2 sequence, preceded by one or more units of monomer 1, is indicated by [*P*_n_^2*^].

The mole fraction of *M*_2_ incorporated into the polymer (*F*_2_), which represents the instantaneous copolymer composition, is defined as follows^69^

7 $$F_{2}=\frac{\left[M_{2}\right]}{r_{1}\left[M_{1}\right]\left(1-\alpha \right)+M_{2}\left(2-\alpha \right)}$$

where α is obtained by solving the quadratic eq 8.

8 $$f\left(\alpha \right)=\alpha^{2}-\left(1+K_{\mathrm{equiv}}\left[M_{2}\right]+\left(\frac{K_{\mathrm{equiv}}}{r_{2}}\right)\left[M_{1}\right]\right)\alpha +K_{\mathrm{equiv}}\left[M_{2}\right]$$

The parameter *K*_equiv_ = *k*_p22_/*k*_dp22_ ^50^ represents the propagation-depropagation equilibrium constant for *M*_2_. It is worth mentioning that the instantaneous copolymer composition depends on the propagation and depropagation rate coefficients as well as the individual monomer concentrations rather than simply their ratio. The influence of these parameters on the estimation of reactivity ratios and on the shape of the instantaneous composition versus comonomer ratio curve will be discussed in detail in the Section 3. The material balances of the monomers for the Lowry case I expressed as the rate of monomer consumption with respect to total conversions, are presented below^66^

9 $$\frac{d\left[M_{1}\right]}{d\mathrm{Xt}}=-\frac{{(\left[M_{1}\right]}_{0}+{\left[M_{2}\right]}_{0})}{1+\left(\frac{\left[M_{2}\right]\left(\frac{1}{1-\alpha }\right)}{r_{1}\left[M_{1}\right]+\left[M_{2}\right]}\right)}$$

10 $$\frac{d\left[M_{2}\right]}{d\mathrm{Xt}}=-\frac{{(\left[M_{1}\right]}_{0}+{\left[M_{2}\right]}_{0})}{1+\left(\frac{r_{1}\left[M_{1}\right]+\left[M_{2}\right]}{\left[M_{2}\right]\left(\frac{1}{1-\alpha }\right)}\right)}$$

The cumulative composition of DBI was calculated as a function of the individual conversions of *M*_1_ and *X*_1_, as follows

11 $$Y_{2}=\frac{\left({\left[M_{2}\right]}_{0}-\left[M_{2}\right]\right)}{\left({\left[M_{1}\right]}_{0}\right)+{\left[M_{2}\right]}_{0})X_{T}}$$

The reactivity ratios *r*_1_ and *r*_2_ were estimated using a parameter estimation algorithm that minimizes the objective function of eq 12 on the cumulative composition as recommended by IUPAC.^70^

12 $$J=\sum_{i=1}^{N}\sum_{j=1}^{\mathrm{Pi}}{\left(Y_{2,i,j}^{\exp}-Y_{2,i,j}^{\mathrm{cal}}\right)}^{2}$$

where *Y*_2_^exp^ are the experimental measurements of a cumulative composition referred to as DBI determined by in situ ^1^H NMR and *Y*_2_^cal^ is the theoretical cumulative composition calculated using eqs 9–11 and the initial comonomer concentrations. *i* refers to the experiment number, while *j* denotes the sample number within each experiment used in the estimation procedure. The reactivity ratios are the estimation parameters in the model. The parameter estimation algorithm was coded in MATLAB using the ODE45 solver to solve ordinary differential equations and LSQNONLIN for nonlinear data fitting. 95% confidence intervals were calculated using the nonlinear regression parameter confidence interval function (NLPARCI).

## 3 Result and Discussion

### 3.1 Copolymerization of DBI with Conventional Monomers (BA/MMA) and Biobased Monomer (2-OA/IBOA)

The solution copolymerizations of BA/DBI were performed at different monomer molar ratios of 50:50, 75:25, and 25:75 in the NMR tube. The conversion evolution of each monomer and the overall conversion over time are presented in Figure 3a–c, for the three different monomer ratios: 75:25 (circle), 50:50 (square), and 25:75 (triangle).

**Figure 3:**
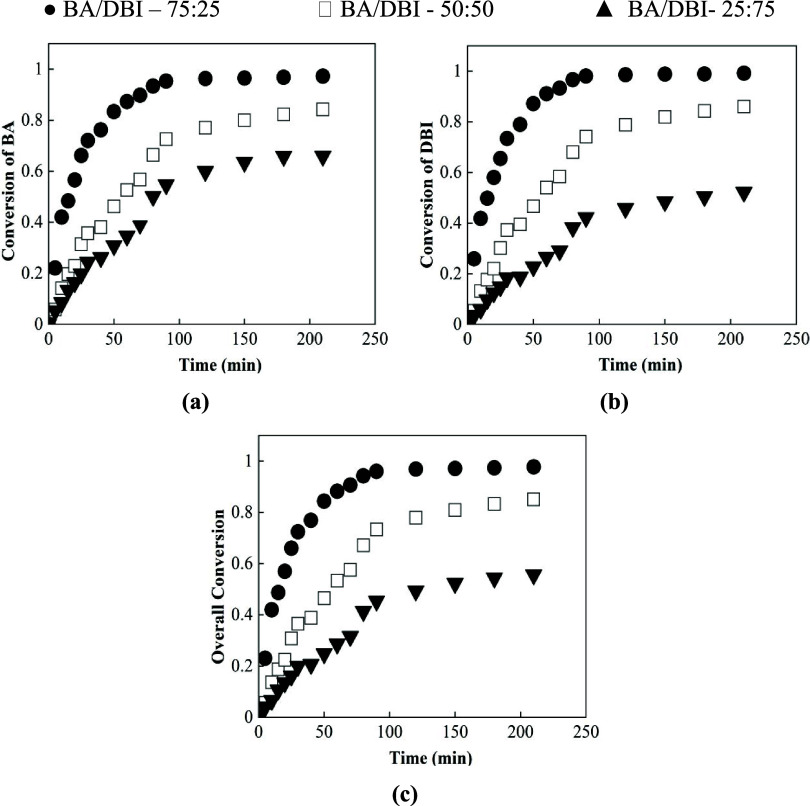
Individual conversion evolution over time of (a) BA and (b) DBI and (c) the overall conversion with the different initial comonomer ratios of 75:25 (circle), 50:50 (square), and 25:75 (triangle).

It can be seen in Figure 3 that individual and overall conversions gradually increase, as expected across the three monomer ratios, demonstrating that BA/DBI monomers copolymerize at comparable rates. Nonetheless, it is noted that for both monomers with a 75:25 ratio, the overall conversion reached values above 95%, whereas at a 25:75 ratio, the conversion was relatively lower, in the range of 50–60%. These results demonstrate that the overall polymerization rate decreases as the DBI fraction increases.

The copolymerization reactions of MMA/DBI were conducted under the same conditions as those used for the BA/DBI system, maintaining the same initial monomer composition ratios. The time evolution of both comonomer conversions and the overall conversion for each of the three experiments with different initial comonomer ratios can be seen in Figure 4a–c. A similar decreasing trend in the polymerization rate with an increasing DBI molar fraction, as observed with BA, can be seen here, but it is even more pronounced. As the DBI fraction in the initial comonomer ratio increases, the overall polymerization rate decreases further. However, in contrast to the BA/DBI copolymerization, MMA shows higher conversions compared to DBI, indicating a higher reactivity ratio of MMA compared to the DBI.

**Figure 4:**
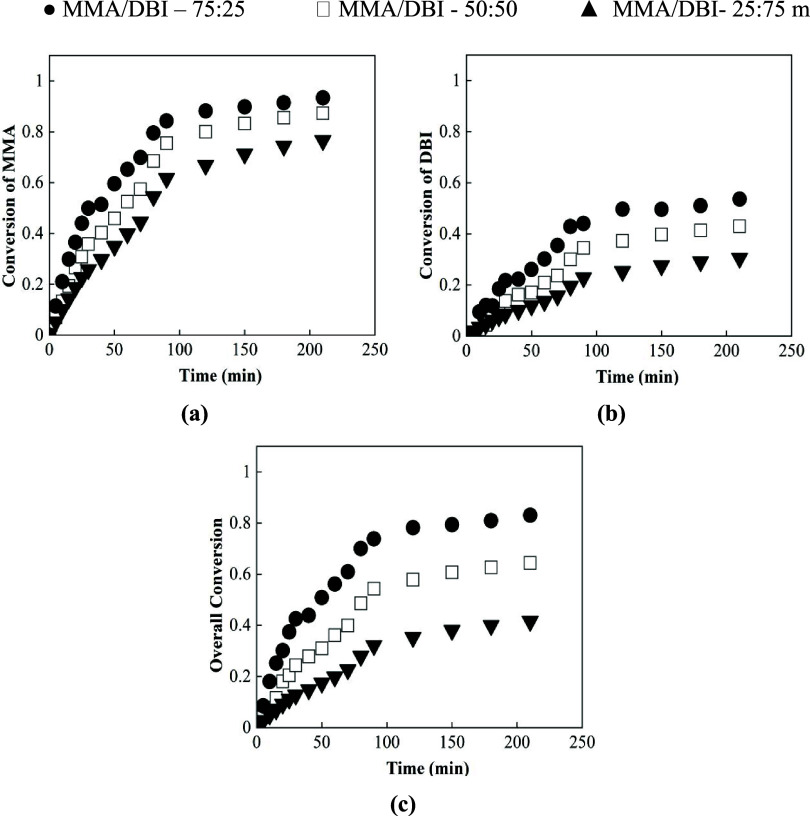
Individual conversion evolution over time of (a) MMA and (b) DBI, and (c) the overall conversion with the different initial comonomer ratios of 75:25 (circle), 50:50 (square), and 25:75 (triangle).

For monomer ratios of MMA/DBI 50:50 or 75:25, conversions above 80% were measured for MMA, whereas conversions around 50% were measured for DBI. However, when 75% of DBI was used, the MMA conversion was decreased, obtaining final values below 80 and 30% for the DBI, which means that by the end of the experiment DBI was not fully incorporated into the copolymer, even at a low initial comonomer ratio.

In the case of copolymerization of 2-OA and IBOA with DBI, the conversion evolution for the two biobased systems of each monomer and the overall conversion over time are presented in Figure 5a–5f, for the three different monomer ratios. Both biobased acrylate systems exhibit copolymerization behavior analogous to that of the BA/DBI system, with high conversions when the ratio was 75:25 acrylate/DBI. Notably, reduced conversion of 50 and 40% was observed when the DBI molar ratio was increased in the initial comonomer ratio, a trend consistently observed across all the studied systems.

**Figure 5:**
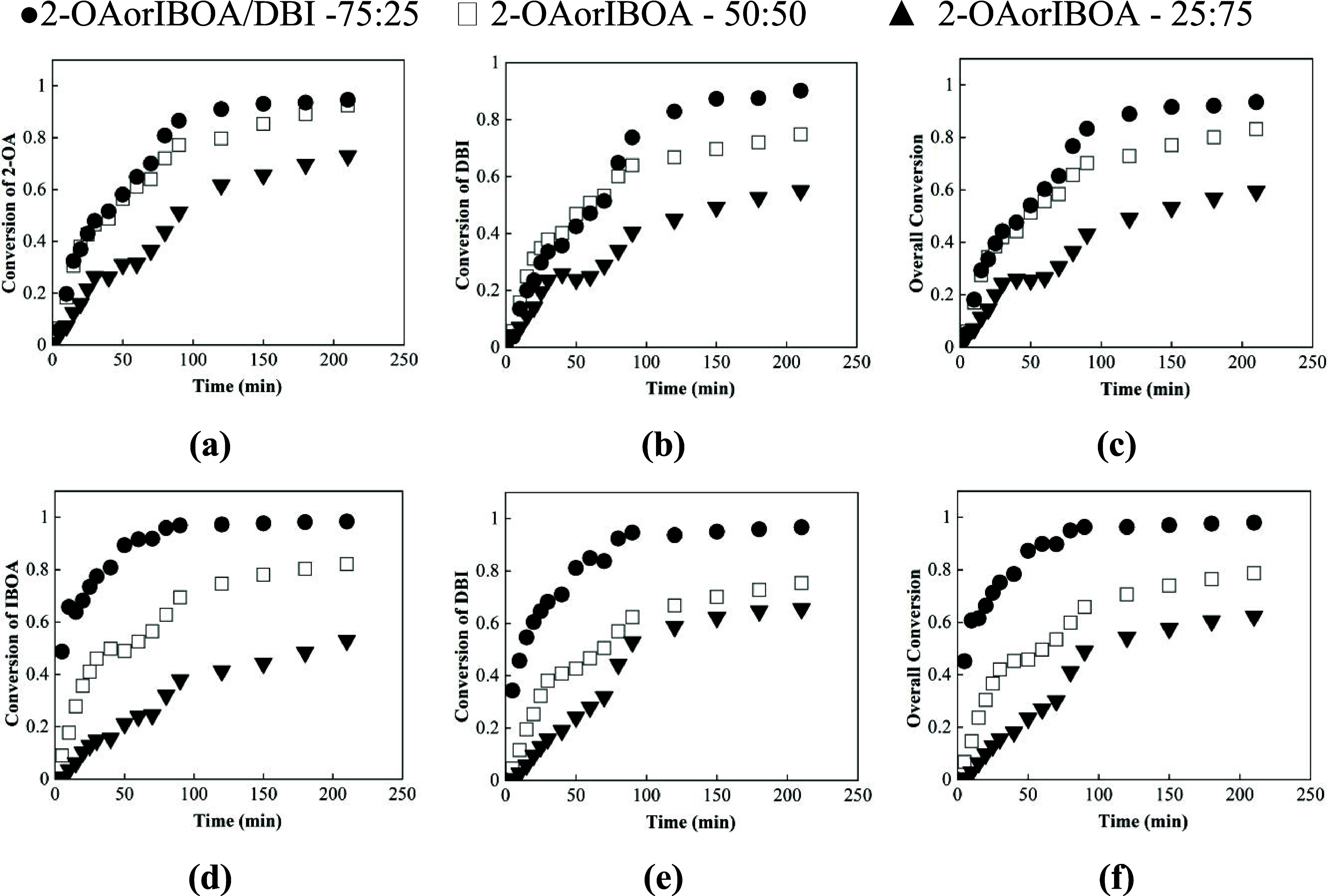
Individual conversion evolution over time of (a) 2-OA and (b) DBI; and (c) the overall conversion. Following similar logic, (d–f) for IBOA-DBI with the different initial comonomer ratios of 50:50 (square),75:25 (circle), and 25:75 (triangle).

It is important to note that all four systems exhibit a common trend: as the DBI content increases, the conversion becomes limited. This limitation is directly linked to depropagation of the DBI monomer.

### 3.2 Estimation of Reactivity Ratio

The nonlinear least-squares (NLLSQ) method explained in Section 2.3 was employed to determine reactivity ratios by analyzing experimental data on the mole concentration of the unreacted monomers throughout the overall conversion for all four copolymerization systems.

As discussed above, when estimating the reactivity ratios of a comonomer that at least one exhibits a tendency for depropagation, the equilibrium rate coefficient must be taken into account. This consideration makes the reactivity ratios dependent on both propagation and depropagation parameters. In this work, we first investigated the effect of uncertainty in the reported depropagation rate coefficients of DBI on the estimated reactivity ratios for the DBI/BA system. Table 1 summarizes recent studies reporting reactivity ratios for the BA/DBI system, which show noticeable discrepancies. For example, Pirman et al.^53^ reported values of the reactivity ratios at 50 °C for the BA/DBI monomer pair as *r*_BA_ = 0.59 and *r*_DBI_ = 0.76, with both reactivity ratios below one. In contrast, Drache et al.^61^ reported the reactivity ratios of *r*_BA_ = 0.5 and *r*_DBI_ = 1.26, indicating that DBI is significantly more reactive than BA, with a reactivity ratio greater than one. It is worth mentioning that, as listed also in Table 1, Pirman et al.^53^ measured the reactivity ratios at lower temperature without considering the depropagation, while Drache et al.^61^ calculated them at temperatures where depropagation is significant, and the depropagation step was explicitly included in the reactivity ratio estimation. In this work, the depropagation rate coefficient of DBI was considered to be as reported by Szablan et al.^33^ with the *k*_dp_ = 7.2 × 10^10^ exp (−63 300/*RT*). Pirman et al.^53^ estimated the prefactor, fitting the experimental data by almost half and reported *k*_dp_ = 3.46 × 10^10^ exp (−63 300/*RT*). To shed light on the discrepancy reported on the reactivity ratios by different authors, we first studied the sensitivity of the estimated reactivity ratios using different rate coefficients of depropagation.

**Table 1:** Reactivity Ratios for DBI/BA and DBI/MMA Copolymerizations Estimated in This Study and Those Reported in the Literature^a^

study	monomer pairs	temperature (°C)	depropagation	*r*_DBI_	±95% CI	*r*_comonomer_	±95% CI
this work	DBI/BA	70	√	0.68	0.14	0.68	0.11
Priman et al.^53^	DBI/BA	50	×	0.76	0.08	0.59	0.03
Drache et al.^61^	DBI/BA	60 and 80	√	1.26		0.5	
this work	DBI/MMA	70	√	0.38	0.10	3.53	0.55
Madruga et al.^27^	DBI/MMA	50	×	0.71	0.11	1.33	0.09
this work	DBI/2-OA	70	√	1.44	0.15	1.64	0.15
this work	DBI/IBOA	70	√	2.0	0.57	1.87	0.52

a In the estimation of the reactivity ratios reported in this work, the *k*_dp_ value reported by Pirman et al.^53^ was used.

Figure 6 illustrates the comparison between the cumulative copolymer composition with respect to DBI, determined experimentally by NMR analysis (points) and the calculated cumulative copolymer composition (lines) using different rate coefficients of depropagation with the corresponding estimated reactivity ratios. It can be seen that the fitting of the experimental data is reasonably good with completely different reactivity ratios. Interestingly, it can be seen that the estimated values in this work (*r*_BA_ = 0.68 ± 0.11 and *r*_DBI_ = 0.68 ± 0.14) using a lower depropagation rate coefficient reported by Pirman et al.^53^ are closer to the values of reactivity ratios measured without considering the depropagation at lower temperatures estimated by the same group, with reactivity ratios below 1. On the other hand, the values estimated using the higher reported *k*_dp_,^33^ which was used in the work of Drache et al.^61^ led to values similar to the ones reported in that work (*r*_BA_ = 0.71 ± 0.12 and *r*_DBI_ = 1.36 ± 0.47). This highlights the strong sensitivity of the reactivity ratio calculations to the depropagation rate coefficient, which must therefore be carefully considered when estimating reactivity ratios.

**Figure 6:**
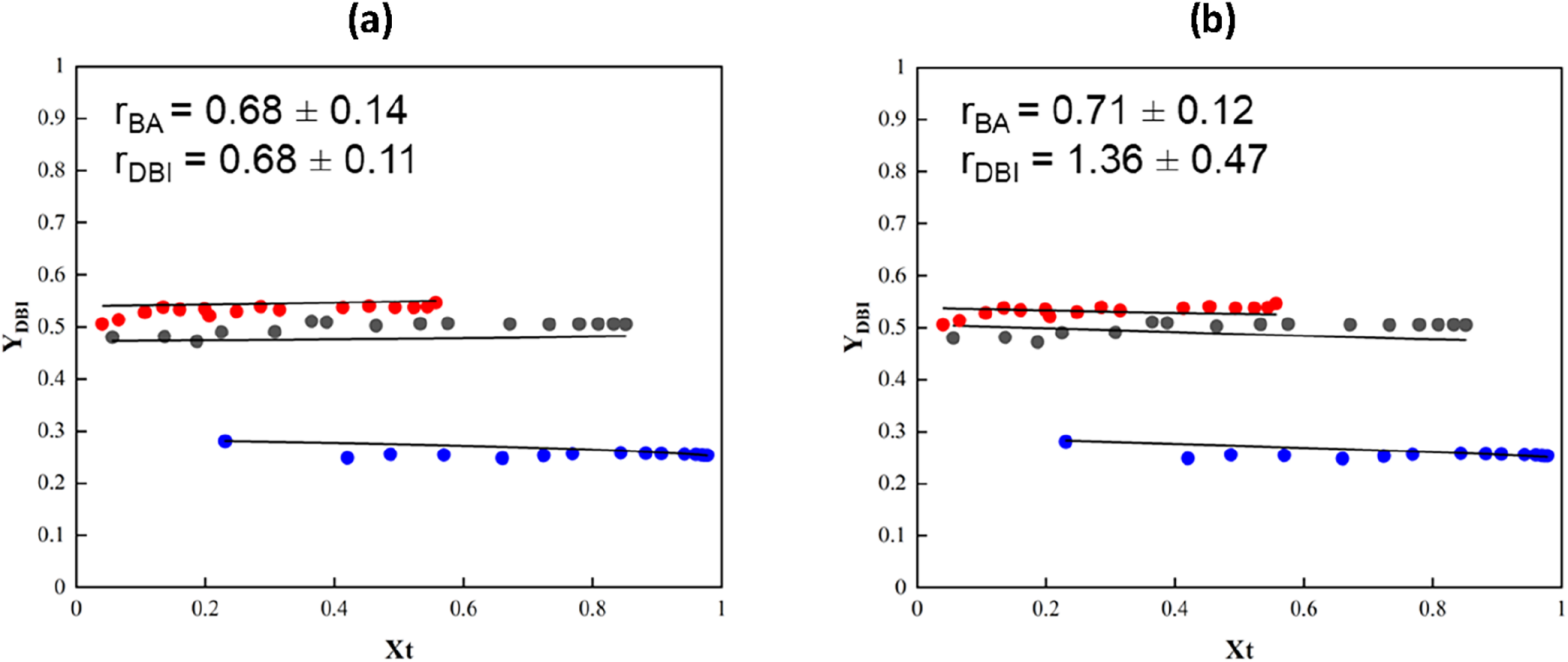
Cumulative copolymer composition of DBI for the in situ ^1^H NMR experiment of BA/DBI pair: (scatter circles) experimental results and (lines) model predictions with the estimated reactivity ratios. Molar ratios of BA/DBI are 50:50 (black circle), 75:25 (red circle), and 25:75 (blue circle). (a) Using *k*_dp_ reported by Pirman et al.^53^ leading to estimated reactivity ratios of *r*_BA_ = 0.68 ± 0.14 and *r*_DBI_ = 0.68 ± 0.11. (b) Using *k*_dp_ reported by Szablan et al.^33^ leading to estimated reactivity ratios of *r*_BA_ = 0.71 ± 0.12 and *r*_DBI_ = 1.36 ± 0.47.

Because the effect of temperature on the reactivity ratios is expected to be minor, the lower *k*_dp_ reported by Pirman et al.^53^ was considered to be more accurate, as it yielded reactivity ratios that were more consistent with those obtained at lower temperatures. This value was, therefore, used for the calculation and reporting of the reactivity ratios for all comonomer pairs throughout this work.

As explained in Section 2.3, in the presence of a depropagation reaction involving one of the comonomers, the Mayo–Lewis equation must be modified. In revised eq (eq 7), the individual monomer concentrations appear separately rather than as a ratio. Consequently, the shape of the copolymer composition curve will differ at temperatures where depropagation is significant, depending on the overall monomer concentration in the solvent. Therefore, no universal copolymer composition can be defined for each pair of monomer systems under such conditions. On the other hand, as the equilibrium coefficient also appears in this equation, temperature will also have an important effect on the shape of the copolymer composition curve at temperatures at which the depropagation is relevant. Figure 7 shows the theoretical instantaneous copolymer composition, *F*_DBI_, as a function of the initial comonomer ratio, *f*_DBI_, using eq 7 and the estimated reactivity ratios in this work calculated at the overall monomer concentration of 1.7 mol/L (corresponds to 30% solids content) and at a temperature of 70 °C. It is worth mentioning that incorporating depropagation into the Mayo–Lewis equation significantly alters the evolution of the instantaneous composition with respect to the initial comonomer ratio. In cases in which an azeotropic point is present, it also shifts the initial comonomer ratio at which the azeotropic point occurs, as shown in Figure S4. In addition, the experimental instantaneous copolymer compositions were calculated using the time evolution of the fractional conversions of each monomer. The polymerization rate of each monomer at each sampling time is determined by the derivative of its fractional conversion. Consequently, copolymer composition, *F*_DBI_, can be easily calculated as follows (eq 13)­

13 $$F_{\mathrm{DBI}}=\frac{R_{p\mathrm{DBI}}}{R_{p\mathrm{DBI}}+R_{p\mathrm{BA}}}$$

**Figure 7:**
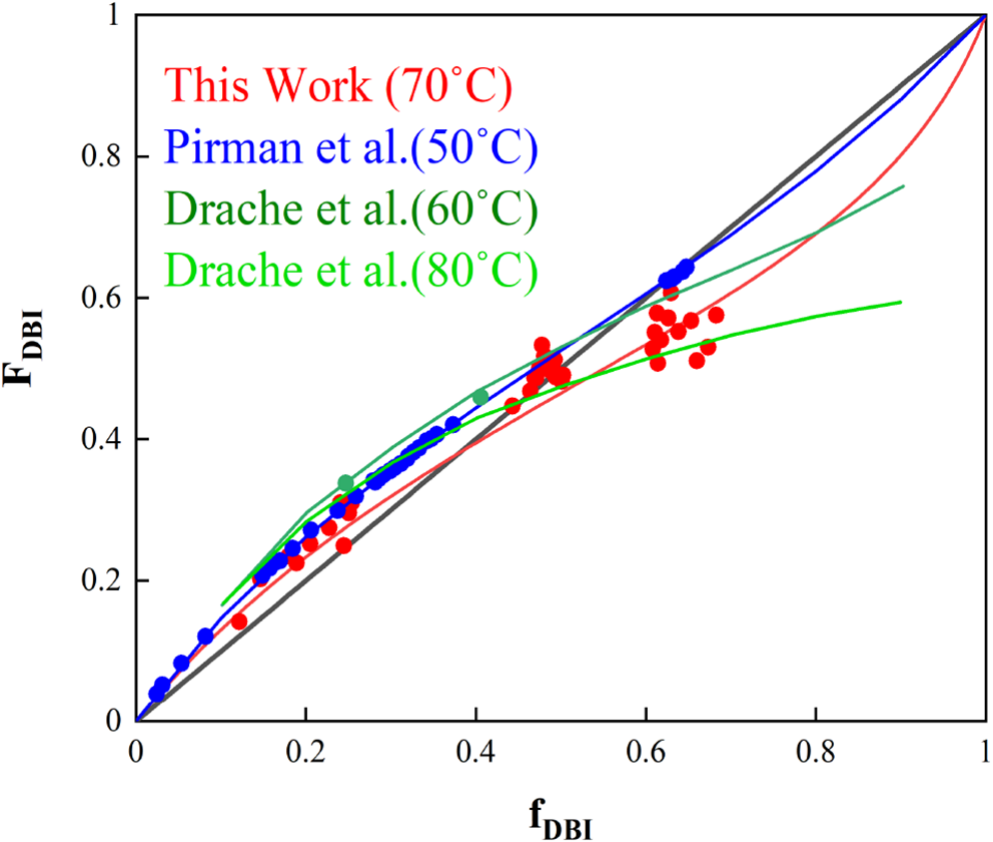
Comparison of the theoretical (lines) and experimental (circles) instantaneous copolymer composition of DBI: using reactivity ratios from this work at 70 °C and 1.7 molar concentration (red) calculated by eq 7(red). Reported by Pirman et al.^53^ at 50 °C (blue). Reported by Drache et al.^61^ at 60° and 80 °C (green).

It can be seen that the set of reactivity ratios estimated in this work not only fits the cumulative copolymer composition reasonably well but also provides a good fit for the instantaneous copolymer composition of DBI (Figure 7).

For comparison, the theoretical instantaneous composition of DBI using the reactivity ratio values reported by Pirman et al.^53^ and Drache et al.^61^ with their corresponding experimental data is plotted as well in Figure 7. It should be mentioned that the data from this work were calculated using the modified Mayo–Lewis eq (eq 7) while the conventional Mayo–Lewis equation was used to calculate the theoretical instantaneous copolymer composition as reported in the work of Pirman et al. In addition, the theoretical data calculated by fixing the itaconate concentration at 0.5 mol/L and determining the corresponding BA concentration (based on the selected initial comonomer ratios using Monte Carlo simulation) and the experimental data by Drache et al.^61^ were taken from the original work. Further details can be found in the cited publications. It can be seen that at low molar fraction of DBI (below *f*_DBI_ = 0.4), all three sets of the reactivity ratios predict comparable instantaneous copolymer composition, while some discrepancies appear at higher DBI molar fractions. It is worth mentioning that the experimental instantaneous composition provided in the work of Drache et al.^61^ does not cover a broad range of molar fraction of DBI, being only reliable at low molar fractions.

Regarding the MMA/DBI system, Figure 8a shows the comparison between the cumulative copolymer compositions for the MMA and DBI copolymer system. It can be seen that the fitting is reasonably good using the estimated reactivity ratios. The reactivity ratios of the monomers, with 95% confidence intervals, are determined to be *r*_MMA_ = 3.53 ± 0.55 and *r*_DBI_ = 0.38 ± 0.10. These results indicate that MMA copolymerizes significantly faster than DBI, as shown by the conversion evolution curves in Figure 4, leading to a clear composition drift during the copolymerization. A similar trend in composition drift was reported by Madruga et al.,^27^ who conducted copolymerization at 50 °C. However, their estimated reactivity ratios (*r*_MMA_ = 1.329 ± 0.09 and *r*_DBI_ = 0.717 ± 0.11) showed a smaller difference between them. Despite the differences in the estimated reactivity values between the two studies, the re-evaluated data in this work show composition drift that aligns with Madruga et al.’s findings.

**Figure 8:**
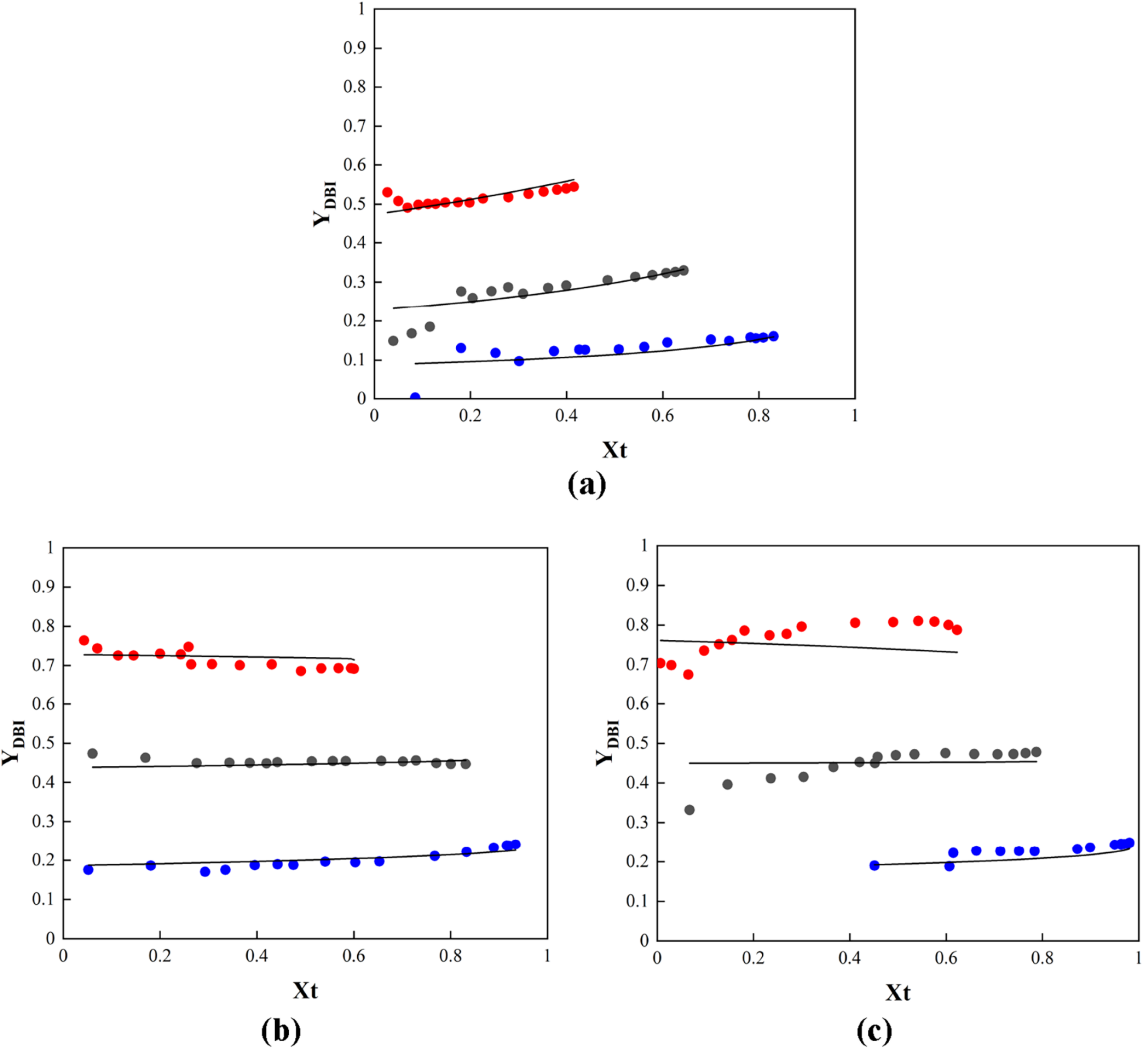
(a) Cumulative copolymer composition of DBI for the in situ ^1^H NMR experiment of the MMA/DBI pair. (b) Cumulative copolymer composition of DBI for the in situ ^1^H NMR experiment of the 2-OA/DBI pair. (c) Cumulative copolymer composition of DBI for the in situ ^1^H NMR experiment of the IBOA/DBI pair.

Figure 9 illustrates the relationship between the initial comonomer ratio composition and the instantaneous copolymer composition of DBI, calculated using the modified Mayo–Lewis eq (eq 7) with the reactivity ratios estimated in this work at 70 °C and a total monomer concentration of 1.97 mol/L. It can be seen that the set of estimated reactivity ratios in this work provides a good fit to both the cumulative and instantaneous copolymer compositions of DBI across a broad range of DBI molar fractions. For comparison, Figure 9 also shows the theoretical instantaneous copolymer compositions of DBI calculated using the Mayo–Lewis equation without considering the depropagation and corresponding experimental data reported by Madruga et al. ^27^ at 50 °C. It can be observed that the set of reactivity ratios estimated by Madruga et al.^27^ does not adequately capture the experimental instantaneous composition of DBI obtained in this study. The impact of temperature (in the range of 50–70 °C) and overall monomer concentration (from 1.97 to 3 mol/L) is minor on the evolution of the instantaneous copolymer composition over the initial comonomer ratio composition, as shown in Figure S5. It can be seen that neither increasing the temperature nor the monomer concentration results in trends similar to those reported by Madruga et al. Therefore, the differences observed between the two studies cannot be attributed to the effects of temperature or monomer concentration in solution polymerization.

**Figure 9:**
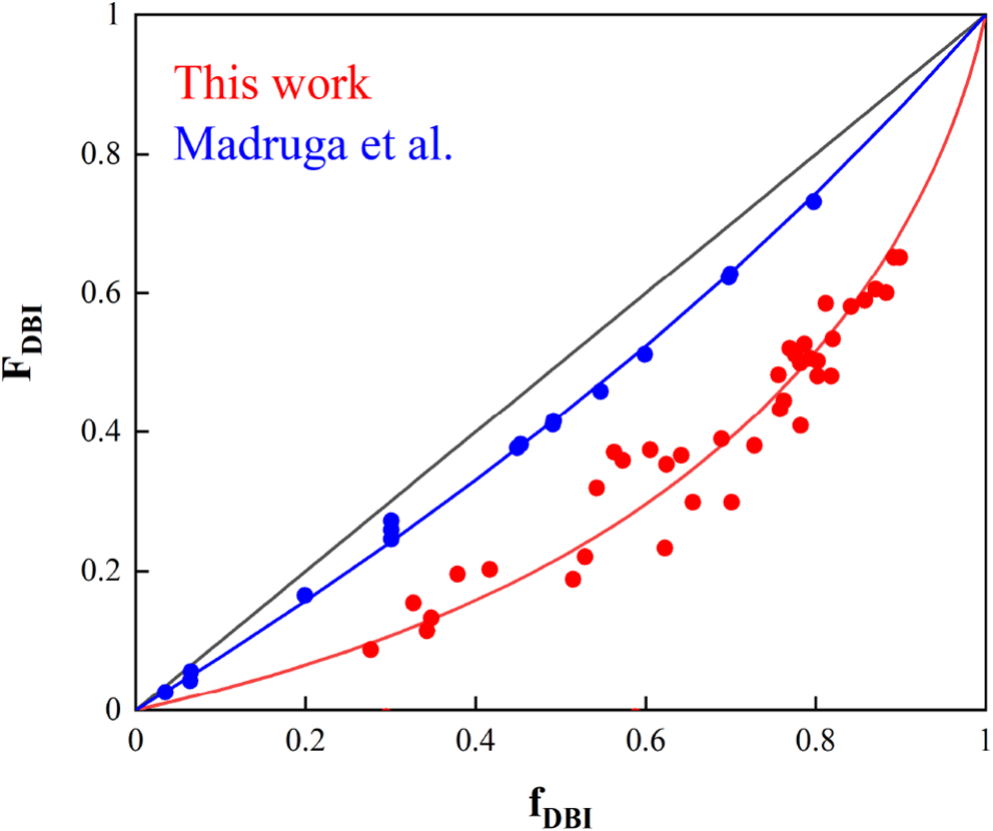
Comparison of the theoretical (lines) and experimental (circles) instantaneous copolymer composition of DBI: using reactivity ratios from this work at 70 °C and 1.97 molar concentration (red) calculated by eq 7 (red). Reported by Madruga et al.^27^ (blue).

In the case of the two copolymerizations carried out using biobased monomers, 2-OA/DBI and the IBOA/DBI systems, Figure 6c,6d illustrate the experimental cumulative copolymer compositions determined by ^1^H NMR (points) and the calculated cumulative compositions using the estimated reactivity ratios (lines). As for the conventional MMA and BA monomers, the alignment between the experimental data and calculated compositions is reasonably good across the overall copolymer conversion.

The estimated reactivity ratios for the 2-OA/DBI system are *r*_2‑OA_ = 1.64 ± 0.15 and *r*_DBI_ = 1.44 ± 0.15, while for the IBOA/DBI system, *r*_IBOA_ = 1.87 ± 0.52 and *r*_DBI_ = 2.0 ± 0.57, as shown in Table 1. It can be seen that for both systems, the estimated reactivity ratios are greater than 1, which is uncommon in free-radical copolymerization. In addition, typically monomers from the same family exhibit similar reactivity ratios. However, these values should be interpreted with caution for systems in which depropagation plays a role since, as shown previously for BA, the estimated reactivity ratios are highly sensitive to the assumed depropagation rate coefficient. In the present work, in the Lowry case I model, it is assumed that two consecutive depropagating monomer units in the polymeric radical can depropagate with the same rate coefficient as the corresponding homopolymer and that the effect of the penultimate unit on this rate coefficient is neglected. Compared to BA, however, IBOA contains a bulky, rigid isobornyl group and 2-OA is branched at the α-position, both of which may influence the depropagation rate of the DBI radical when these monomers are present in the prepenultimate position. It is therefore important to interpret the reactivity ratios in conjunction with the specific depropagation rate coefficient used for their estimation. As illustrated in Figure S6 of the Supporting Information for the DBI/2-OA system, using a significantly lower depropagation rate coefficient leads to different estimated reactivity ratios.

Figure 10 displays the theoretical instantaneous copolymer composition with experimental data for the 2-OA/DBI and IBOA/DBI systems calculated at 70 °C and the overall monomer concentrations of 1.63 and 1.52 mol/L, respectively. The experimental instantaneous copolymer composition was calculated from the individual conversion curves. It can be seen that in both cases, the estimated sets of reactivity ratios provide a good fit not only for the cumulative copolymer composition but also for the instantaneous copolymer composition. In a copolymer system where both reactivity ratios are above or below one, an azeotropic composition might be found. Even if in Figure 10, this composition is not appreciable, there exist high DBI monomer compositions at 0.97 and 0.99 for the 2-OA and IBOA system, respectively. Precisely, it has been observed that the addition of the depropagation effect in the Mayo–Lewis equation shifted this azeotropic point toward larger values of the *f*_DBI,_ as it can be seen in Figure S4 for both 2-OA/DBI and IBOA/DBI.

**Figure 10:**
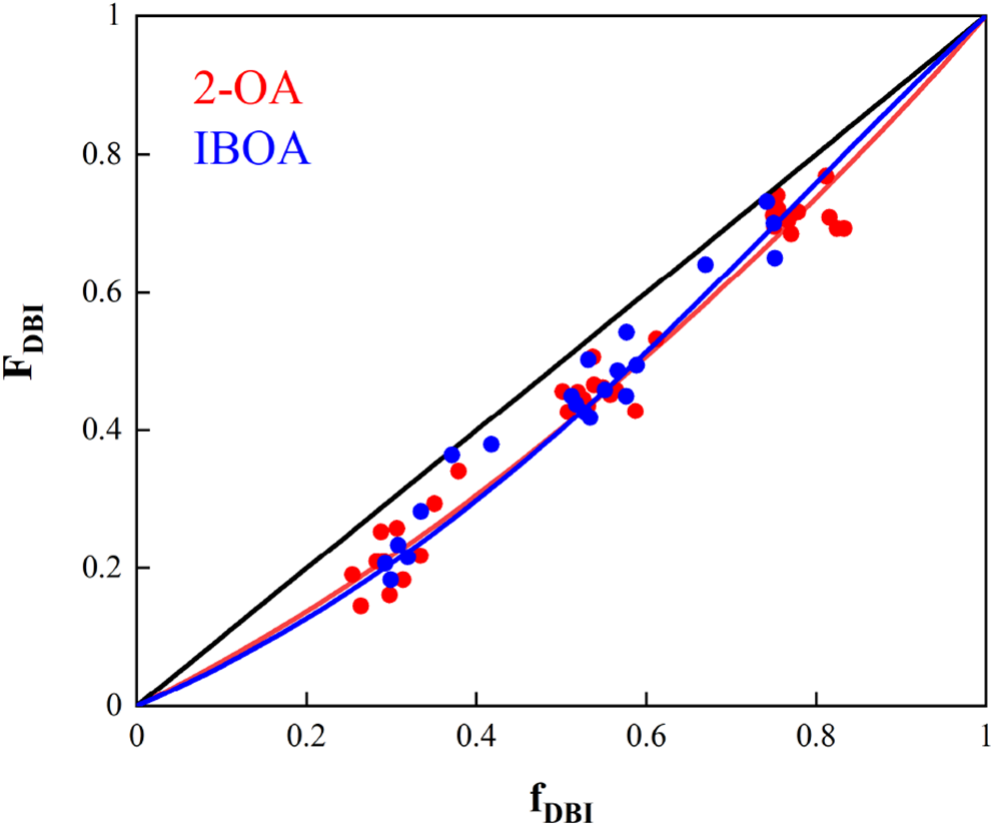
Theoretical instantaneous copolymer composition was calculated using the reactivity ratios by incorporating depropagation according to Lowry Case I (red line). The experimental instantaneous copolymer composition (red circles) was determined from the evolution of the individual monomer conversions obtained for 2-OA/DBI. Similarly, the theoretical (blue line) and experimental (blue circles) instantaneous copolymer compositions for the IBOA/DBI system were determined in the same manner.

## 4 Conclusions

Polymeric derivatives of itaconates represent a promising class of monomers derived from renewable resources. These monomers hold significant potential for copolymerization with various comonomers via free-radical polymerization. In this work, the reactivity ratios of biobased dibutyl itaconate (DBI) with both conventional and biobased meth­(acrylates) were estimated using the nonlinear least-squares method, taking into account the depropagation of DBI.

The effect of the depropagation rate coefficient on the BA/DBI system was examined to compare the discrepancies between the values obtained in this work and those reported in the literature. It was found that the estimated reactivity ratios are highly sensitive to the depropagation rate coefficient, which must therefore be carefully considered in estimating reactivity ratios. For the BA/DBI system, both reactivity ratios were estimated to be below one, using the depropagation rate coefficient reported by Pirman et al., indicating that cross-propagation is favored over homopropagation.

Using the same depropagation rate coefficient of DBI, for the biobased acrylate monomers and DBI systems (2-OA/DBI and IBOA/DBI), both estimated reactivity ratios were found to be above one, which is uncommon, suggesting that the prepenultimate effect on the DBI depropagation rate coefficient should be considered in such systems with bulky comonomers. In the case of the MMA/DBI system, the reactivity ratios were found to be *r*_MMA_ = 3.53 ± 0.55 and *r*_DBI_ = 0.38 ± 0.10, indicating that this system exhibited the greatest composition drift among those studied. A comparison with previously reported reactivity ratios from the literature is also provided.

This study demonstrates that both conventional and biobased acrylates are excellent candidates to address the inherently slow radical polymerization rate of DBI, as their incorporation significantly enhances the overall reaction rate. Leveraging these reactivity data enables the design of high biocontent copolymer compositions that are well-suited for industrial applications, offering improved sustainability and expanding the use of biobased materials.

## Supplementary Material



## Notes

^1^H NMR spectra of MMA-DBI; ^1^H NMR spectra of 2-OA-DBI; ^1^H NMR spectra of IBOA-DBI; effect of temperature and molar concentration on the theoretical instantaneous copolymer composition of DBI in the DBI/MMA copolymerization; and comparison of the theoretical evolution of the instantaneous copolymer composition with and without accounting for depropagation (PDF)
