# Supplementary material for: Reactivity Ratios of Biobased Dibutyl Itaconate with Conventional and Renewable (Meth)Acrylates: Influence of Depropagation
Source: Biomacromolecules. 2025 Oct 13;26(11):8074–86. doi: 10.1021/acs.biomac.5c01505 (PMC12606552; doi:10.1021/acs.biomac.5c01505)
Supplement: Supplementary file 1 [file bm5c01505_si_001.pdf]

Supporting Information

Reactivity Ratios of Bio-Based Dibutyl Itaconate with Conventional and Renewable (Meth)Acrylates: Influence of Depropagation

Jyoti Gupta[1], Radmila Tomovska[1,2], Shaghayegh Hamzehlou[1] and Miren Aguirre*[1]*

[1]POLYMAT, Kimika Aplikatua Saila, Kimika Fakultatea, University of the Basque Country UPV-EHU, Joxe Mari Korta Zentroa, Tolosa Hiribidea 72, 20018 Donostia-San Sebastián, Spain.

[2]IKERBASQUE, Basque Foundation for Science, Plaza Euskadi 5, 48009, Bilbao, Spain

Results and Discussion

NMR spectra

The monomer conversions of MMA and DBI were measured following the evolution of the peaks of the protons at the carbon atoms and MMA (δ [ppm] = 6.02) and DBI (δ [ppm] = 6.21) as indicated in Figure S1. The NMR spectra for the copolymerization of MMA/DBI and the change of intensity of the peaks over polymerization time can be seen in Figure S1.

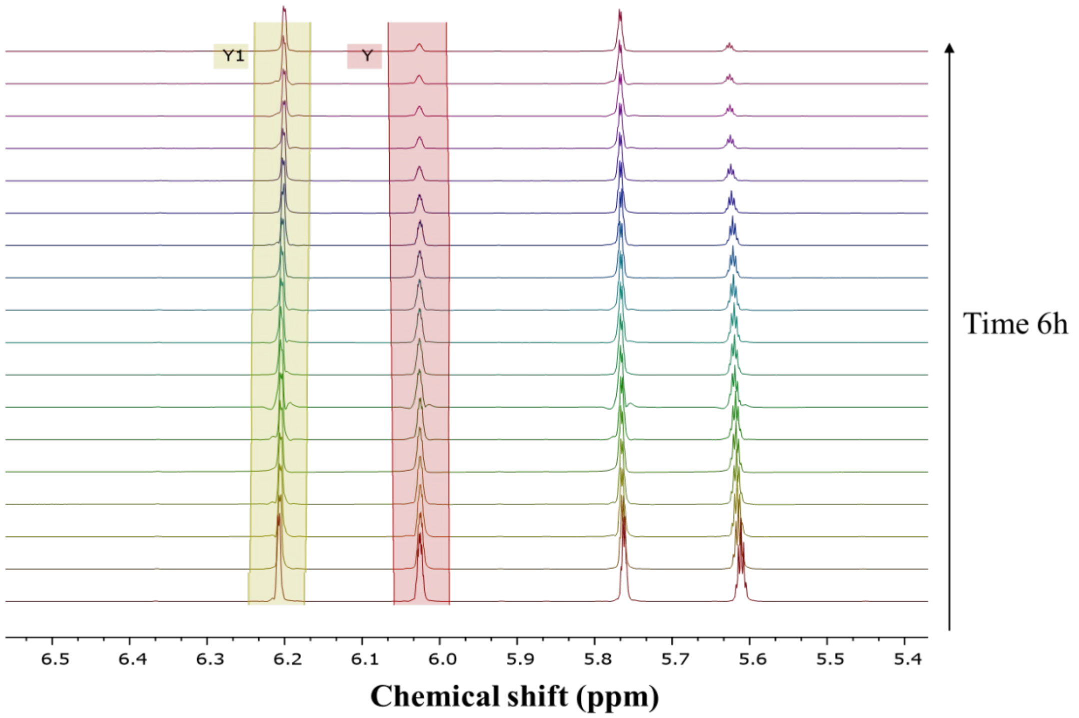

Figure S1: Time evolution of the proton nuclear magnetic resonance (^{1}H-NMR) spectra of the solution copolymerization of MMA and DBI carried out at 70˚Cin a 50/50 ratio. The peaks that were monitored to follow the conversion of MMA are marked in red (Y) and the ones for DBI are marked in yellow (Y1).

The monomer conversions of 2-OA and DBI were measured following the evolution of the peaks of the protons at the carbon atoms and 2-OA (δ [ppm] = 5.85-5.91) and DBI (δ [ppm] = 5.76) as indicated in Figure S2. The NMR spectra for the copolymerization of 2-OA/DBI and the change of intensity of the peaks over polymerization time can be seen in Figure S2.

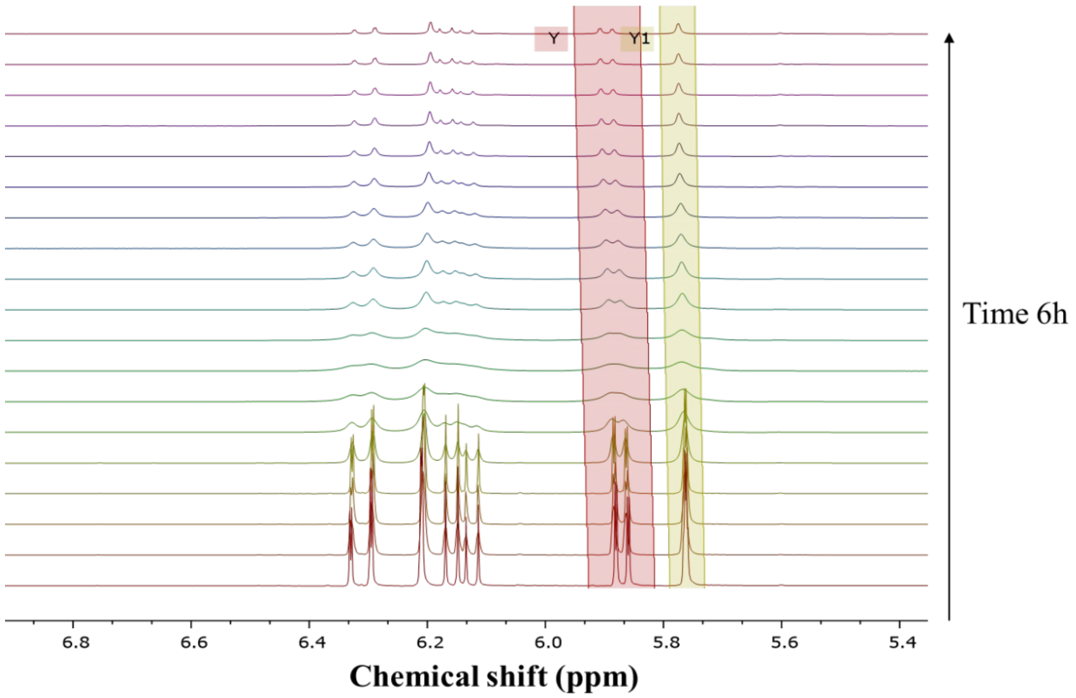

Figure S2: Time evolution of the proton nuclear magnetic resonance (^{1}H-NMR) spectra of the solution copolymerization of 2-OA and DBI carried out at 70˚C in a 50/50 ratio. The peaks that were monitored to follow the conversion of 2-OA are marked in red (Y) and the ones for DBI are marked in yellow (Y1).

The monomer conversions of IBOA and DBI were measured following the evolution of the peaks of the protons at the carbon atoms and IBOA (δ [ppm] = 5.85-5.91) and DBI (δ [ppm] = 5.76) as indicated in Figure S3. The NMR spectra for the copolymerization of IBOA/DBI and the change of intensity of the peaks over polymerization time can be seen in Figure S3.

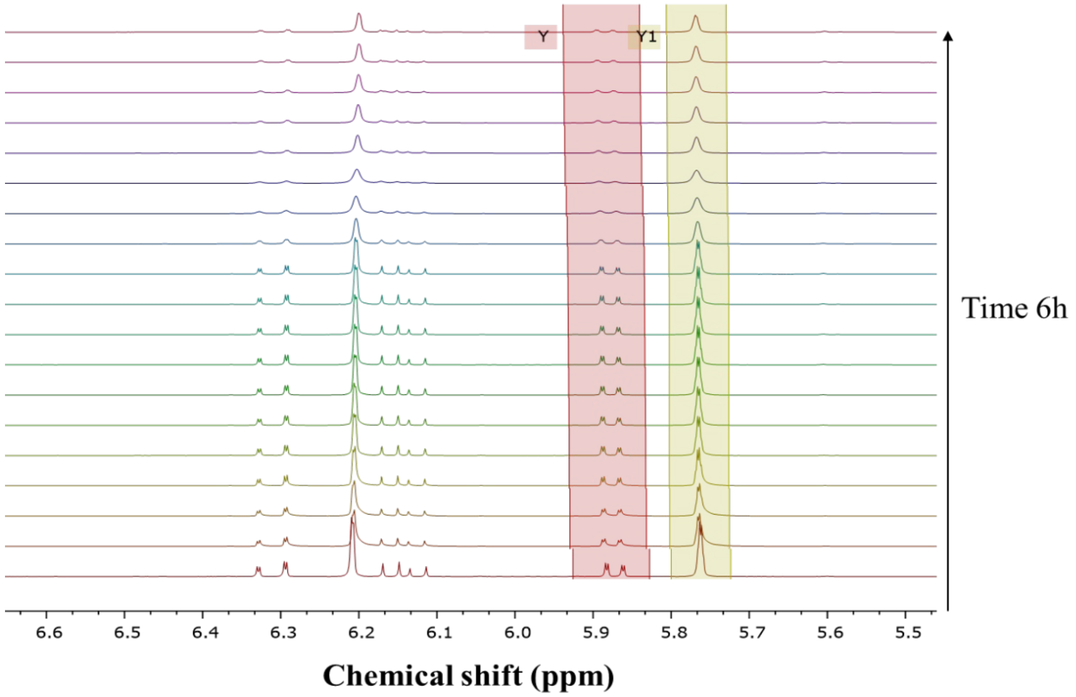

Figure S3: Time evolution of the proton nuclear magnetic resonance (^{1}H-NMR) spectra of the solution copolymerization of IBOA and DBI carried out at 70˚C in a 50/50 ratio. The peaks that were monitored to follow the conversion of IBOA are marked in red (Y) and the ones for DBI are marked in yellow (Y1).

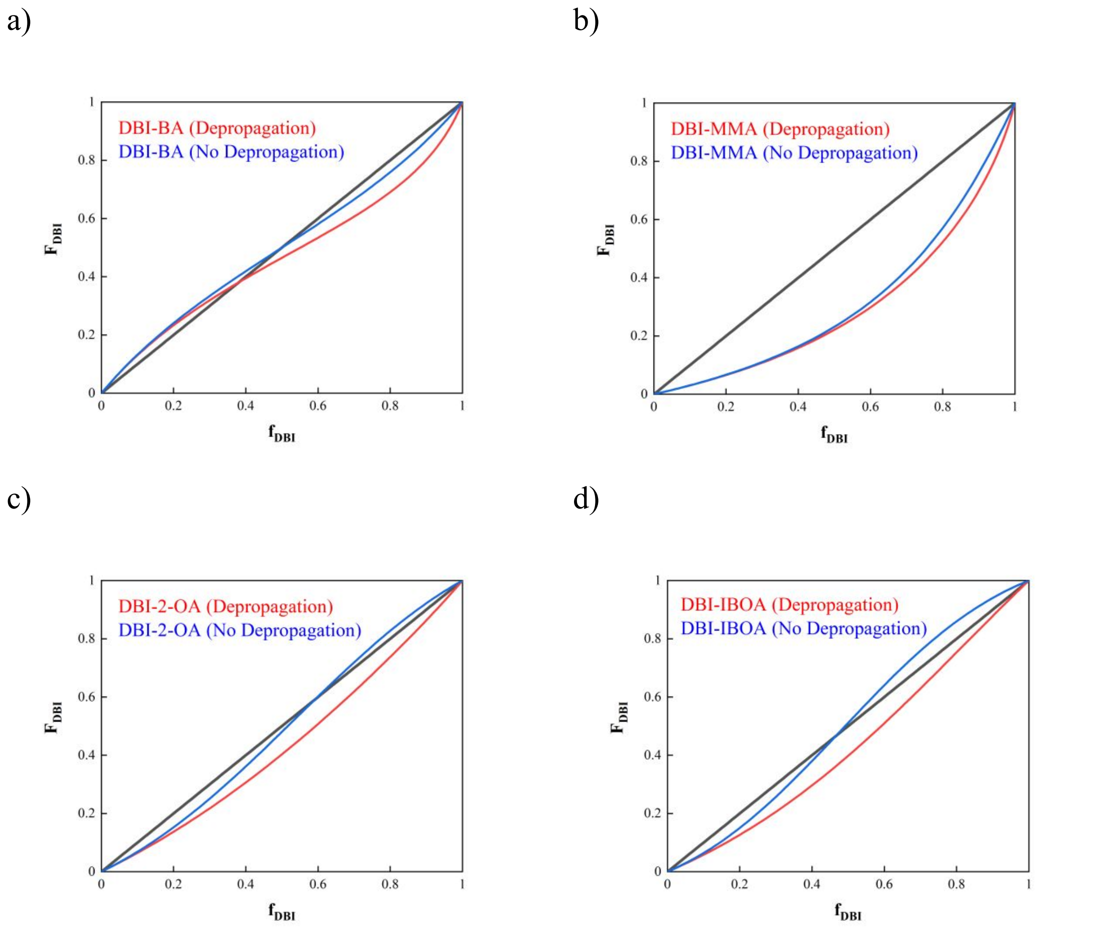

Figure S4. Comaprison of the evolution of instantanous copolymer compostion with and without accounting for depropagation for a) BA/DBI, b) MMA/DBI, c) 2-OA/DBI and d) IBOA/DBI copolymer systems, using the estimated reactivity ratios in this work.

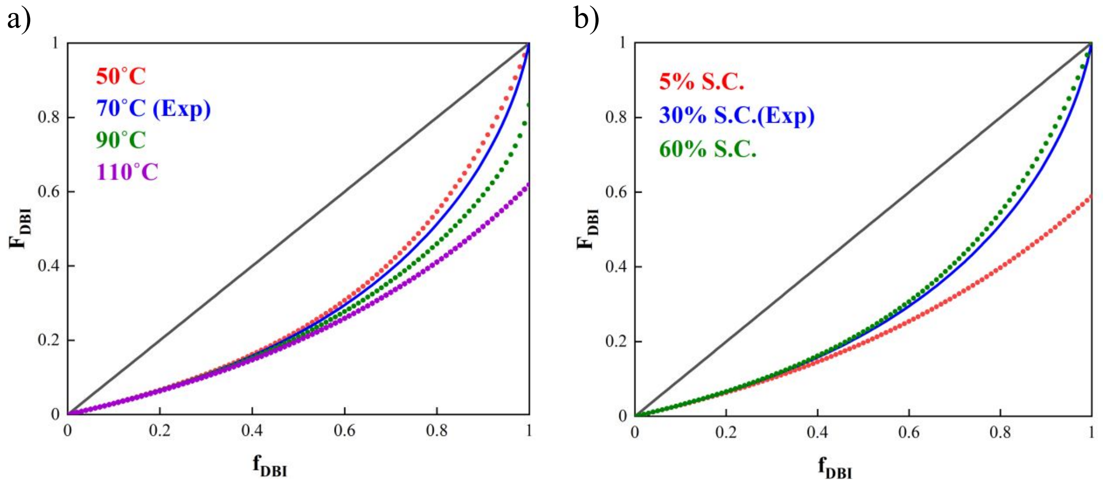

Figure S5. Effect of a) temperature (calculated at 30% S.C.), b) solids content (calculated at 70 ˚C) on the theoretical instantaneous copolymer composition of DBI in the DBI/MMA copolymerization.

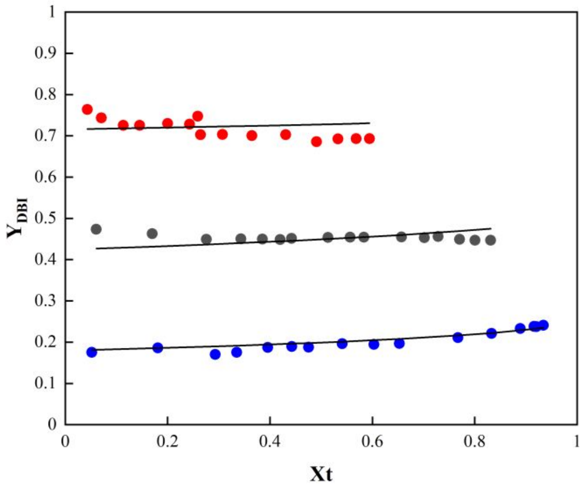

Figure S6. Cumulative copolymer composition of DBI for the in situ ^{1}H-NMR experiment of 2-OA/DBI pair: (scatter circles) experimental results, and (lines) model predictions with the estimated reactivity ratios. Molar ratios of 2-OA/DBI are 50/50 (black circle), 75/25 (red circle), and 25/75 (blue circle) using k_{dp} two orders of magnitude lower than the one reported by Pirman et al. [53] leading to estimated reactivity ratios of $r_{2\text{-}OA} = 1.68 \pm 0.22$ and $r_{DBI} = 0.97 \pm 0.12$.
